# Some Difficult Cases and Their Treatment

**Published:** 1888-08

**Authors:** L. P. Haskell

**Affiliations:** Chicago.


ARTICLE IV.

### SOME DIFFICULT CASES AND THEIR TREATMENT.

BY L. P. HASKELL, CHICAGO.

[Read before the Illinois State Dental Society.]

Were the conditions of all mouths similar and equally favorable, the insertion of artificial dentures would be a mere pastime to the experienced dentist. But unfortunately for both patient and dentist this is not the case, as we all have good reason to know. It is often difficult to make the patient comprehend this, as she remarks that " my mother (or some other intimate friend) has a set of teeth she can scarcely pull out of her mouth, and she can even crack nuts with them and why cannot I ?"

Often the work is undertaken without considering all the unfavorable conditions of the mouth ; for while some mouths are so favorable that a set of teeth made by a novice will work well, others are so unfavorable that the utmost care and experience are necessary to construct them ; the patient will have to exercise patience, and require a much longer time to learn to use them.

As long as I have been engaged in a practice exclusively prosthetic, new combinations still present themselves ; and some of them are very difficult to decide as to what is best to be done to secure satisfactory results.

After a careful diagnosis of such cases, I explain to the patient the conditions of the case, and endeavor to make clear its difficulties and what may reasonably be expected from the work when completed. Often, not the least difficult factor is expense. Either the patient cannot or will not submit to the expense necessary to secure the best results, especially when a metal plate is a *sine vua non.* This is sometimes true of persons who, in all other things, procure the *best* regardless of expense. As for instance, a lady who was absolutely suffering from the non-conductibility of rubber plates, so much so that she was often compelled to remove them to relieve her mouth from the heat, remarked " I am not going to pay so much as that for a set of teeth ;" and yet on her person were thousands of dollars worth of diamonds. She, however, came back two months later and had the teeth made, and said to me afterward,"no money could buy them if I could not get another set like them."

Sometimes patients, after being told what must be done to secure the best results, will allow you to complete a part of the work and then refuse to have the rest done because perhaps some tooth or root must be removed, and then complain that the work is not satisfactory, as the following case will illustrate:

A lady consulted me as to what should be done with her mouth, apparently willing to abide by my judgment. There were a few loose teeth and roots in the upper jaw, and anterior teeth and a few roots in the lower. I advised the extraction of all the upper and all the roots in the lower, and the insertion of an upper and partial lower. She consented, and I extracted the upper; but her courage failed, and she would not allow me to remove the lower roots. I made her a temporary upper, and impressed upon her the necessity sooner or later for a partial lower. She wore the tempory a year, and returned for her permanent. She still objected to the extraction of the lower roots. I made the permanent upper and told her she would have trouble with them unless she had lower posterior teeth to sustain the plate when the mouth closed, for the plate would be constantly displaced. The upper maxillary was very thin, and the lower teeth somewhat prominent, so that they would close outside the arch. Had it been a receding lower jaw, the teeth closing well inside, the upper teeth would have been in a measure supported. The upper set was all right in every respect, but the closure would diplace it. The result has been dissatisfaction and complaint to all her neighbors; though I sent her word that if she would let me do as I wished, and then the upper troubled her after a fair trial, I would make no charge for the work, so confident was I of success. But the conditions are even worse now than when the teeth were made, for the constant pressure on the front has caused complete absorption of all the process, and she now bites against a yielding ridge.

Another class of cases is where the upper teeth are all gone, and on the lower jaw the six anterior teeth and one or two bicuspids on one side remain. There being no teeth on the opposite side of the jaw to counterbalance the pressure of these one or two bicuspids, the plate is constantly displaced; for there is no remedy, but the extraction of bicuspids, because artificial teeth on the opposite side would soon yield to pressure and the pressure revert to one side. Extract and insert the partial lower and thus keep the pressure equalized. The extraction of one or two sound teeth subserves the best interests of the patient. This must be regarded rather than mere sentinent.

There are patients where in the upper jaw all the anterior and on one side all the posterior teeth are missing. In such cases the patient would be far better off if they were all out, still I hesitate to advise it, but do the next best thing, viz., make a suction plate, and put a clasp on one of the bicuspids to counteract the pressure on the other side in masticating; at the same time let the pressure be on the natural teeth.

Then we find patients in whose mouths all the lower teeth, and one-half or two-thirds of the upper are missing —from the central incisors back. Such a case I had a year ago. This is a worse condition than the one last mentioned for the lower artifical meet on one side natural teeth and unyielding pressure, and on the other side artificial and yielding pressure. Here is a case, again, where the only effectual remedy is the extraction of the remaining upper teeth; a partial remedy is secured by shortening the lower teeth, every few months, on the side of the upper natural, to relieve the excessive pressure.

Frequently where the palatal bone is prominent, forming an arch, and extending far back, if "vacuum cavities" are ever deemed necessary they should always be avoided in these cases, as they interfere with the success of a plate. Simply raise the plate entirely clear of it, but allow it to extend beyond at that point, far enough to find a resting place.

When the upper jaw is very prominent and the lip short, and the patient shows teeth and gums, often quite high, the artificial gum must be very thin, high and without seams. There is here but one method of supplying a denture that will fulfill all the requirements; that is by the use of continuous-gum work. With this the teeth can be set under the margin of the gums; the porcelain gum can be made thin as desired and yet strong, as it is baked to the plate, and you have a perfect denture. Some may say, use a gold or rubber plate and set the teeth (plain) against the margin of the gums. But you will have a weak denture, and one that is not as firm as if the plate was carried over the outside of the process. Besides, there is no mouth where the cuspid teeth have been extracted a year or more, that there is not an absolute necessity for making the gum fuller and higher at those points than elsewhere, and this can in such cases be done only with this style of work.

When the upper teeth and lower anterior teeth remain and the patient requires masticating surface, the insertion of a partial lower set is necessary. In doing this, the attempt is often made to relieve the pressure and wear of the anterior teeth by making the artificial teeth sufficiently long. This is always a mistake; the pressure of natural teeth on the posterior artificial teeth results in constant discomfort till the gums have yielded to the pressure so as to let the teeth down sufficiently to allow the anterior teeth to meet again. Better let them meet at this point at first, and if the anterior teeth have become shortened by wear, "shoe" them.

In many interspersed partial cases the pressure is allowed to bear on the artificial teeth when it should, if possible, be left on the natural teeth, thereby preventing excessive pressure on narrow surfaces of gum.

I wish to emphasize the vast importance of the word *articulation* as applied to the closure of artificial teeth. More depends on it than anything connected with the success of artificial dentures. Many a denture, right in every other particular, is entirely wrong in this, and consequently a source of discomfort to its wearer.

Three rules cover essentially the ground. Never allow pressure on the six anterior teeth; never, in full upper plates, allow the pressure to be greater on one side than the other; never allow a second or third lower molar, which has projected forward so that its face shows, to meet an artificial tooth at that angle, as it will surely crowd forward the upper plate, the same as the meeting of the anterior teeth.

As a rule, a full lower plate is more comfortable and useful than a partial, because the pressure is distributed equally over the whole jaw.

ARTICLE V.

